# Increased Insulin Concentrations During Growth Hormone Treatment in Girls With Turner Syndrome Are Ameliorated by Hormone Replacement Therapy

**DOI:** 10.3389/fendo.2020.586055

**Published:** 2020-12-14

**Authors:** Sabine Elisabeth Segerer, Stephan Georg Segerer, Carl-Joachim Partsch, Wolfgang Becker, Frank Nawroth

**Affiliations:** ^1^ Department of Endocrinology, Centre for Infertility, Prenatal Medicine, Endocrinology and Osteology, Amedes Hamburg, Hamburg, Germany; ^2^ Department of Nephrology, Asklepios Barmbek, Hamburg, Germany; ^3^ Department of Pediatrics, Endokrinologikum Hamburg, Hamburg, Germany; ^4^ Department of Laboratory Medicine, Medizinisches Versorgungszentrum (MVZ) MediVision Altona GmbH, Hamburg, Germany

**Keywords:** hyperinsulinemia, obesity, cholesterol, Homeostasis Model Assessment-Insulin Resistence (HOMA-IR)****, Body Mass Index (BMI)

## Abstract

**Objective:**

Turner syndrome (TS) is characterized by complete or partial loss of one sex chromosome and is commonly associated with short stature, metabolic changes (such as central obesity, abnormal glucose tolerance and high triglycerides) and premature ovarian insufficiency (POI). Primary management of TS during childhood and adolescence comprises treatment with human growth hormone (hGH) and, in cases with early loss of ovarian function, hormone replacement therapy (HRT). Given that metabolic parameters are altered when HRT is applied during menopause, we analyzed whether metabolic changes might be positively or negatively affected within 10 years after HRT and/or hGH in girls with TS.

**Design:**

Observational study.

**Methods:**

Data were collected from the medical records of 31 girls with TS attending two endocrinologic centers in Germany between 2000 and 2020. Descriptive statistics are reported as the mean ± SEM or percentages.

**Results:**

The mean age at first presentation was 99.06 ± 8.07 months, the mean height was 115.8 ± 3.94 cm, and the mean BMI 19.0 ± 0.99 was kg/m^2^. Treatment with hGH was given to 96.8% of the girls, starting at an average age of 99.06 ± 8.70 months, and was continued for 67.53 ± 6.28 months. HRT was administered to 80.6% of all patients and was started at a mean age of 164.4 ± 4.54 months. During the follow-up, we did not observe any significant absolute changes in lipid parameters, but we detected beneficial effects of childhood hGH: significantly lower cholesterol (-0.206/month; p = 0.006), lower low density lipoprotein cholesterol (-0.216/month; p = 0.004), and higher high density lipoprotein cholesterol (+0.095/month; p = 0.048). Insulin concentrations, showed a significant increase attributable to hGH treatment (+0.206/month; p = 0.003), which was ameliorated by concomitant or subsequent HRT (-0.143/month; p = 0.039).

**Conclusion:**

Treatment with hGH and HRT is provided to most girls with TS. Metabolic effects are associated with both modalities. Monitoring of metabolic changes appears to be important to detect unfavorable effects, and could guide treatment adjustment and duration.

## Introduction

Turner syndrome (TS), one of the most common sex-chromosome abnormalities, affects approximately 1 in 2,500 newborn female infants (1). It is caused by complete or partial loss of one X chromosome. The spectrum of phenotypes broadly differs depending on the karyotype (45,X, 45,X/46,XX mosaicism and structurally abnormal X) (2–4). A main feature of almost all girls with TS is decreased growth leading to lower adult height (95–100%) (5). Thus, primary management during childhood and adolescence focuses on appropriate growth through established treatment with human growth hormone (hGH) (6). Beyond affecting growth, hGH has important effects on metabolism. In a cross sectional study, hGH-treated girls with TS have been found to have significantly lower abdominal adiposity and better glucose tolerance than untreated girls with TS (7). Other studies investigating the effects of hGH treatment have found that prior hGH treatment positively influences lipid parameters (8, 9) and decreases the prevalence of arterial hypertension (10), thus potentially decreasing the risk of cardiovascular events.

Beyond growth restriction effects, most adolescents with TS exhibit increased gonadotropins over time, with low estradiol concentrations indicating premature ovarian insufficiency (POI) (90–95%) (5). Consequently, spontaneous pregnancies are only rarely observed (11). To date, accelerated loss of oocytes during fetal development is thought to induce an early increase in gonadotropins (12). Although POI is common, the timing varies and depends on the karyotype. Patients with 45,X/46,XX mosaicism have been found to have greater rates of spontaneous puberty and menarche (13). However, even those individuals often develop POI in subsequent years (4). Anti-Müllerian hormone (AMH) has been found to be in the reference range in all patients with Turner mosaicism and in 43% of those with miscellaneous karyotypes such as structural abnormalities. Independently of the karyotype, the concentrations of AMH are relatively constant in females 8 to 25 years of age (14).

In girls with early loss of ovarian function, hormone replacement therapy (HRT) is applied to induce puberty. Induction of puberty is usually started at 11–12 years (6). A meta-analysis of the effects of estrogen replacement therapy on women with TS has shown that oral estrogen replacement therapy is linked to a greater increase in high density lipoprotein cholesterol (HDL-C) than transdermal therapy. Other lipid fractions are not affected beneficially (15). To date, whether cardiovascular outcomes are ameliorated by HRT in patients with TS is unknown.

Central obesity is common in women with TS (16). Compared with age-matched controls, women with TS have higher BMI and greater central adiposity (16). Additionally, abnormal glucose tolerance and high triglycerides (TG), which are also observed during natural menopause, are found in patients with TS (17).

Physiological menopause transition is also accompanied by weight gain and changes in body composition (18). Studies in mice have indicated that oophorectomy decreases energy expenditure and promotes an increase in adipose tissue independently of diet (19). Supplementation with 17β estradiol in ovariectomized female mice protects against adipocyte hypertrophy, as well as adipose tissue oxidative stress and inflammation (20). In humans, hormonal changes during menopause are also associated with a significant increase in visceral adipose tissue (VAT) (21, 22). This increase in VAT is associated with insulin resistance and a higher prevalence of metabolic syndrome, which are risk factors for cardiovascular diseases (23). HRT appears to be associated with decreased overall fat mass, improved insulin sensitivity and lower rates of type 2 diabetes (18). However, the beneficial effects of HRT do not persist after discontinuation of therapy (24). Another study focusing on atherogenic lipid profiles in postmenopausal women has shown a significant decrease in plasma concentrations of cholesterol, low-density lipoprotein cholesterol (LDL-C) and Lipoprotein a after HRT for 3 months compared with placebo (25).

Hypothesizing that HRT might also affect metabolic parameters in girls with TS, we investigated the metabolic changes in the context of concomitant/prior hGH treatment in girls with TS over a follow-up period up to 10 years. Additionally, we analyzed whether patients with initially high FSH (> 20 mIU/ml) might develop a higher BMI or poorer metabolic parameters, similarly to the weight gain observed in postmenopausal women.

## Materials and Methods

### Data Collection

**Figure 1:**
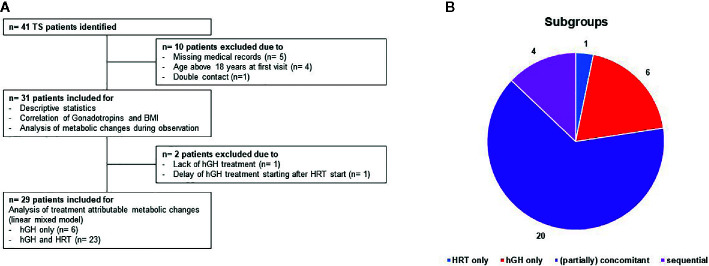
**(A)** Flowchart of the study period including an overview of drop outs and therapy regimens. 41 patients with TS have been identified. Thereof, 10 patients have been excluded due to missing medical records (n = 5), age above 18 years at first visit (n = 4) or double contact (n = 1) leaving 31 for descriptive statistics, correlation of gonadotropins and BMI and analysis of metabolic changes during observation. For analysis of the attribution of hGH/HRT to metabolic changes (linear mixed model), another two patients have been excluded due to lack of hGH treatment or delayed hGH treatment. Panel **(B)** shows the distribution of therapies within the study population: 1 patient HRT only (blue color), 6 patients hGH only (red), 4 patients sequential therapy (hGH following HRT) (pink), and 20 patients partially concomitant treatments (purple).

Data were retrieved retrospectively from the medical charts of girls with TS attending two different private, non-university endocrinologic centers (Endokrinologikum Hamburg and Centre for Infertility, Prenatal Medicine, Endocrinology and Osteology Hamburg) from January 2000 to March 2020. A total of 41 patients with TS were identified, whereof one patient has been attending both centers and therefore has been omitted (**Figure 1**). The inclusion criteria were confirmed diagnosis of TS by karyotype, age < 18 years at first presentation and clinical follow-up ≥ 5 years. A total of nine patients were excluded from the study because of either missing medical record information (n = 5) or an age above 18 years at the first visit (n = 4), thus leaving 31 patients for further evaluation. The study was conducted according to the guidelines of the Declaration of Helsinki after formal approval was obtained from the Research Ethics Committee of the Hamburg Medical Board, allowing the use of the anonymized data for research purposes. All patients provided written informed consent to anonymous use of their individual data in the study. The study design is summarized in **Figure 1** (**Figure 1A**: design; **Figure 1B** subgroup of patients on hGH/HRT).

### Clinical Data

Clinical information including age at first presentation, age at spontaneous menarche without a history of HRT, history of HRT (start, composition and stop/non-adherence) and hGH treatment (start and duration), Tanner pubertal staging, coexisting congenital malformations, and endocrine and autoimmune disorders were obtained from the patients’ files. Height and weight measurements were collected, and the BMI and the standard deviation score (SDS) were calculated for every visit. The clinical criterion for starting HRT was hypergonadotropic gonadotropins. In those cases, HRT was started with estradiolvalerat as a liquid (0.4 g/ml, two drops initially, with escalating doses, and an additional two drops every month until a maximum of ten drops was reached). hGH was started at doses of 40–50 µg/kg/day. An increase in dosage was provided depending on the increase in growth and on insulin-like growth factor (IGF-1) concentrations.

### Biochemical Data

During the clinical follow-up, blood samples were routinely collected at every visit. The clinical follow-up was conducted every 3 months during hGH treatment and every 6 months during HRT. Laboratory parameters were collected from the medical records retrospectively from the first visit, with a follow-up of 5 to 10 years. A complete blood count and blood biochemistry tests were performed with standard laboratory methods (**Supplementary Table 1**). Insulin concentrations were measured with IMMULITE ^®^ 2000 immunoassays (DPC Biermann, Germany) from 2000 to 2007; thereafter, analysis was performed with LIAISON^®^ chemiluminescence technology (DiaSorin, Italy) (**Supplementary Table 1**). Glucose and lipid concentrations were measured from 2000 to 2005 with an AU5400 analyzer (Olympus, Germany); thereafter, tests were performed with a Cobas modulator platform (Roche, Germany). Serum follicle-stimulating hormone (FSH) and IGF-1 were measured with electrochemiluminescence immunoassays (Elecsys^®^, Roche Diagnostics). Serum AMH concentrations were determined with Beckman Coulter enzyme immunometric assays until 2018; thereafter, measurement was performed with electrochemiluminescence immunoassays (Elecsys^®^, Roche Diagnostics).

### Statistics

Descriptive statistics are reported as the mean ± SEM or percentages unless indicated otherwise. Statistical analysis was performed in Excel 2010 (Microsoft Corporation, Redmond, WA, USA) and Prism 6 (GraphPad Software Inc., La Jolla, CA, USA). For examination of auxologic and metabolic parameters in the follow-up, Wilcoxon matched pairs signed rank test was performed. To analyze whether initially high FSH (> 20 mIU/ml), similar to that in postmenopausal women, might be associated with poorer metabolic parameters or greater BMI, we compared the BMI of women with initial hypergonadotropic FSH concentrations to those with normogonadotropic FSH concentrations in the follow-up. Kruskal-Wallis test was applied to compare matched pairs. For assessment of metabolic parameters each month, depending on the treatment, onset and duration of the therapy, a generalized linear mixed model was used. The HRT and hGH treatment durations up to a given measurement point, and their interactions, were used as predictors of patient BMI and metabolic parameters. Given the nested structure of the data (four measurement points for each individual), we used a multilevel modeling approach taking this interdependence into account. To account for deviations from the normal distribution assumption, we used a generalized linear mixed model instead of the conventional linear mixed model. We used a two-level approach, with level 1 referring to variations in the key variables (HRT and hGH treatment duration, their interaction, and the criteria BMI, HbA1c, cholesterol, TG, HDL-C, and LDL-C) and level 2 representing stable characteristics of the individual patient. The z-standardized age at therapy onset (either HRT or hGH) served as only a level 2 predictor. For simplicity, age was entered as a linear predictor in our analyses. Given that non-linear effects of age on some of the variables appeared plausible, we re-ran our analyses by using age squared as an additional predictor. The patterns of the results remained the same. Missing values were addressed with the maximum likelihood estimation approach. Statistical significance was defined by p-values < 0.05. The generalized linear mixed model was conducted with the statistical program IBM SPSS 25.

## Results

### Descriptive Characteristics

Regarding karyotypes, 16 (51.6%) had a monosomy (45,X), 6 (19.4%) had a mosaicism (45,X/46,XX), and 9 (29%) had miscellaneous karyotypes (46,XX del; 46X i(X) q10; 45X/46XX/47 XXX;45,X/46,XX/46,X,idic Xq).

The mean age at first presentation was 99.06 ± 8.70 months. The prevalence of spontaneous puberty and menarche was higher in patients with miscellaneous karyotypes (1/9; 11.1%) than with karyotype 45,X0 (0/16; 0%), and was highest in patients with 45,X/46XX mosaicism (3/6; 50%).

Congenital malformations were detected in 40.7% of all patients (cardiac, renal, or ear). Associated autoimmune and endocrine disorders were found in 21.9% of all patients. Neurocognitive and psychosocial issues (emotional immaturity, learning disorders, or behavioral problems) were reported in 12.5% of girls with TS.

### hGH Treatment and HRT

**Figure 2:**
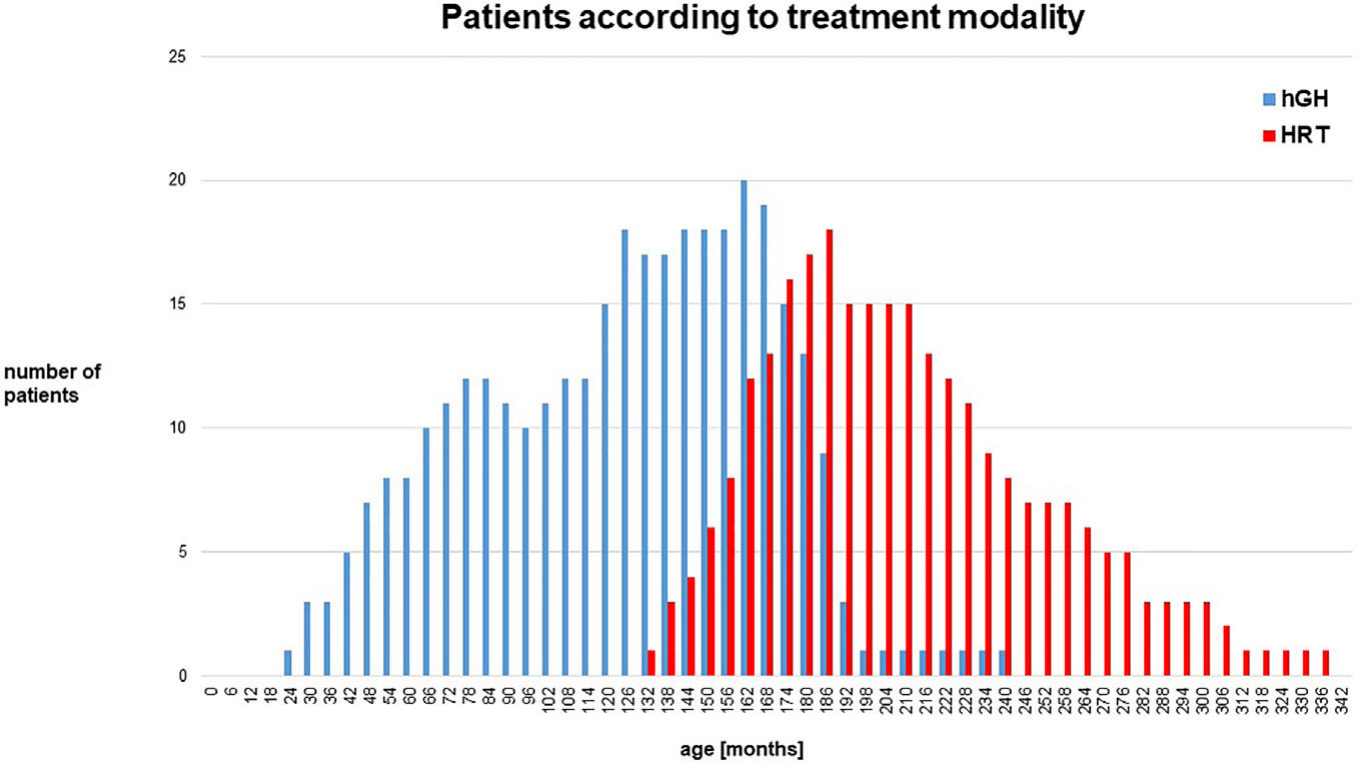
Depicts the number of patients during the follow-up on the different treatment modalities. Blue lines: number of patients on hGH treatment only, red lines: number of patients on HRT at the age of 24–336 months.

The initial IGF-1 concentration (baseline, mean) was 204 ± 31.4 ng/ml, and the initial patient height was 115.8 ± 3.94 cm, which was below the mean average (SDS -1.99 ± 0.19). A significant gain in height SDS was observed in the follow-up (**Table 1**; p = 0.001). In contrast, the initial BMI SDS was on the mean, although we observed a non-significant increase in BMI SDS. **Figure 2** summarizes the numbers of patients on hGH/HRT during the follow-up. Treatment with hGH was given to 96.8% of the girls, starting at an average age of 99.06 ± 8.70 months (mean starting dose: 0.045 mg/kg/day) and was continued for 67.53 ± 6.28 months (last dose mean: 0.040 mg/kg/day).

**Table 1:** Auxologic and metabolic changes of girls and women with TS were analyzed by Wilcoxon matched pairs signed rank test.

Parameters [ranges]	Baseline	Last visit	P
Age	99.06 ± 8.70	217.5 ± 10.34	p < 0.0001	
Height (cm)	115.8 ± 3.94	152.0 ± 1.79	p < 0.0001	
Height SDS	-1.99 ± 0.19	-1.06 ± 0.16	p = 0.001	
Weight (kg)	28.24 ± 3.18	59.14 ± 3.28	p < 0.0001	
Weight SDS	-0.49 ± 0.25	0.19 ± 0.26	p = 0.0007	
BMI (kg/m^2^)	19.02 ± 0.99	25.24 ± 1.18	p < 0.0001	
BMI SDS	0.33 ± 0.25	0.72 ± 0.21	ns	
HbA1c (%)	5.1 ± 0.08	5.45 ± 0.24	ns	
Insulin [mIE/L]	8.29 ± 1.57	20.26 ± 3.11	0.0103	
HOMA-IR	1.59 ± 0.35	5.07 ± 0.92	0.0029	
Cholesterol [mg/dl]	175.2 ± 4.16	168.1 ± 5.43	ns	
LDL-C [mg/dl]	92.25 ± 4.51	93.14 ± 5.74	ns	
HDL-C [mg/dl]	60.96 ± 2.62	64.48 ± 4.18	ns	
TG [mg/dl]	110.4 ± 14.76	105.4 ± 12.22	ns	

Data are reported as mean ± SEM. Statistical significance was defined by p-values < 0.05. ns: non-significant.

HRT was administered to 80.6% of patients in total. Thus, the mean age at the start of HRT was 164.4 ± 4.54 months, and the mean duration was 62.84 ± 7.85 months. Induction of puberty/menarche was mainly performed with escalating doses of estradiolvalerate. In 29 patients, an oral form was used, and in two patients, a dermal application was used. A total of 7.4% of patients were recorded in the follow-up as having stopped the HRT.

### Gonadotropins and AMH Concentrations

Initial FSH concentrations (baseline and first visit) ≤12 mIE/ml were detected in 19/31 (61.3%) of the girls: 9/16 (56.3%) with karyotype 45,X, 4/6 with karyotype 45,X/46,XX (66.7%) and 6/9 with miscellaneous karyotype (66.6%). After 5 years, concentrations of FSH ≤ 12 mIE/ml were found in only 1/16 of girls with karyotype 45,X (6.25%), 3/6 of girls with karyotype 45,X/46,XX (50%) and 1/9 of girls with miscellaneous karyotype (11.1%). AMH was documented in only seven patients at the first presentation. In follow-up (10 years) only two patients—both with the 45,X/46,XX karyotype—had detectable AMH (2.47 ± 1.13 ng/ml).

### Correlation Between Gonadotropins and BMI

In both normogonadotropic and hypergonadotropic TS patients, initial BMS SDS were on the mean and did not significantly differ (**Table 2**). Only normogonadotropic patients showed a significant increase in BMI SDS (p = 0.0479) and HOMA-IR (p = 0.023) in the follow-up. Other metabolic parameters (HbA1c, insulin, cholesterol, LDL-C, HDL-C and TG concentrations) did not differ significantly.

**Table 2:** Baseline values of metabolic parameters of girls and women with TS dependent on initial FSH values.

Parameters	FSH < 20mIU/ml	FSH ≥ 20 mIU/ml	p
BMI (kg/m^2^, baseline)	17.76 ± 0.67	20.63 ± 2.31	ns
BMI (kg/m^2^, last visit)	21.79 ± 0.84	24.28 ± 2.13	ns
baseline vs. last visit	(p < 0.0001)	(p = 0.0195)	
BMI SDS (baseline)	0.42 ± 0.22	0.20 ± 0.52	ns
BMI SDS (last visit)	0.89 ± 0.24	0.30 ± 0.44	ns
baseline vs. last visit	p = 0.0479	ns	
HbA1c (%, baseline)	5.06 ± 0.10	5.18 ± 0.12	ns
HbA1c (%, last visit)	5.33 ± 0.18	5.20 ± 0.04	ns
baseline vs. last visit	ns	ns	
Insulin (mIE/l, baseline)	8.99 ± 2.23	6.60 ± 2.20	ns
Insulin (mIE/l last visit)	19.02 ± 3.75	23.53 ± 5.31	ns
baseline vs. last visit	ns	ns	
HOMA-IR (baseline)	1.69 ± 0.48	1.42 ± 0.51	ns
HOMA-IR (last visit)	4.88 ± 1.19	5.40 ± 1.53	ns
baseline vs. last visit	0.023	ns	
Cholesterol (mg/dl, baseline)	174.8 ± 4.50	174.0 ± 8.27	ns
Cholesterol (mg/dl, last visit)	164.2 ± 5.21	161.5 ± 7.33	ns
baseline vs. last visit	ns	ns	
TG (mg/dl, baseline)	112.8 ± 14.95	104.3 ± 30.8	ns
TG (mg/dl, last visit)	89.89 ± 7.69	95.36 ± 14.97	ns
baseline vs. last visit	ns	ns	
HDL-C (mg/dl, baseline)	59.35 ± 2.91	63.33 ± 4.06	ns
HDL-C (mg/dl, last visit)	64.64 ± 5.02	63.00 ± 5.11	ns
baseline vs. last visit	ns	ns	

Kruskal -Wallis test was applied to analyze whether hypergonadotropic or normogondotropic gonadotropins could affect BMI within the follow-up. Data are reported as mean ± SEM. Statistical significance was defined by p-values < 0.05. ns: non-significant.

### Metabolic Changes Attributable to HRT and hGH Treatment

The metabolic profile at the last day of presentation is shown in **Table 1**. HbA1c, cholesterol, TG, HDL-C and LDL-C were not significantly changed in patients in follow-up. Only insulin and HOMA-IR showed a significant increase (**Table 1**). To detect whether the treatment modalities had different effects on the metabolic parameters, we used a generalized linear mixed model of monthly changes controlled by age at onset, duration of therapy and duration of concomitant hormone treatment (hGH + HRT). We detected that the increase in insulin was attributable to hGH treatment (+0.206/month; p = 0.003; **Table 3**) and that it was ameliorated by HRT (-0.143/month; p = 0.039). In the follow-up, no significant changes in lipid parameters were observed. However, we found a significant decrease in cholesterol (-0.206/month; p = 0.006) and LDL-C (-0.216/month; p = 0.004) as well as an increase in HDL-C (+0.095/month; p = 0.048) attributable to hGH treatment.

**Table 3:** Changes of auxologic and metabolic parameters of women with TS per month without therapy (age-related increase), growth hormone treatment, hormone replacement therapy.

Parameters [per month]	Age-related increase	p	hGH-related effects	p	HRT-related effects	P
HbA1c [%]	-0.001	ns	+0.001	ns	+0.003	Ns
Insulin [mIE/l]	+0.116	ns	+0.206	0.003	-0.143	0.039
HOMA-IR	+0.022	ns	+0.054	0.006	0.000	ns
Cholesterol [mg/dl]	-0.058	0.004	-0.206	0.006	+0.200	ns
LDL-C [mg/dl]	+0.008	ns	-0.216	0.004	+0.310	0.020
HDL-C [mg/dl]	-0.078	ns	+0.095	0.048	+0.037	ns
TG [mg/dl]	-0.183	ns	+0.322	ns	+0.402	ns

Data were analyzed by linear mixed model and are reported as mean ± SEM. Statistical significance was defined by p-values < 0.05. ns: non-significant.

## Discussion

In this retrospective observational study, we showed that hGH treatment was associated with beneficial changes in the lipid profile, whereas this effect was not seen for HRT. However, adverse effects of hGH treatment on insulin were ameliorated by concomitant or subsequent HRT.

During childhood and adolescence, a main focus in the management of girls with TS is on improving adult height. For this purpose, treatment with hGH was initiated in 1983 (26). To date, some debate remains regarding the optimal age for initiation of hGH treatment. Accordingly, the documented age at initiation varied broadly (from 9 months to 10.2 years) in a recent meta-analysis (27). In our study, the average age at the start of hGH treatment was at 99.06 ± 8.07 months, in contrast to the current clinical practice guidelines for the care of girls and women with Turner syndrome, which recommend starting at 48–72 months of age (6). Nevertheless, these recommendations did not exist when most of the patients entered this study. However, hGH treatment was applied to most of the girls (96.8%) and continued for 67.53 ± 6.28 months. A recent study has not observed significant differences in hGH treatment duration (≥3 years vs. <3 years) regarding growth and BMI (8). However, longer exposure to hGH in childhood is associated with beneficial long-term effects on lipid metabolism. An earlier study has detected beneficial effects on LDL-C even after 6 months of hGH treatment (9). In our study, we also observed a beneficial effect of hGH treatment, including lower cholesterol, LDL-C, and higher HDL-C concentrations. However, age- and HRT-related effects appeared to abolish these favorable effects during follow-up in our analysis.

Compared with women with 46,XX karyotype, impaired glucose homeostasis has been detected in 25–78% of adult women with TS (17). Those with karyotype 45,X in particular have impaired glucose metabolism (28), possibly because of the deletion of some X chromosome genes associated with insulin signal transduction and β cell function. As in previous studies (29), we also observed a significant increase in insulin, which appeared to be attributable to hGH treatment. In three cases, diabetes mellitus developed during follow-up (all of which involved hGH treatment for more than 72 months). A beneficial effect of concomitant or subsequent HRT on the development of hyperinsulinemia was detected. Studies on postmenopausal women have suggested that HRT prevents the development of diabetes mellitus (30, 31). In women with TS, the data are controversial. Gravholt et al. have found that HRT is associated with higher incidence of insulin resistance in women with TS compared with healthy age-matched women (32), whereas other studies have not shown significant effects on insulin sensitivity after 6 months (33). Given the potent effects of hGH on insulin secretion in girls with TS, monitoring of fasting insulin and glucose concentrations during hGH treatment should be considered. To date, the current guidelines recommend monitoring HbA1c annually regardless of hGH therapy (6).

We did not observe a significant increase in BMI SDS in all girls during the follow-up of 10 years, in contrast with other observations showing a higher BMI and larger average waist circumference in women with TS (34). This difference may be due to the younger age of the patients in our study. Higher BMI values during childhood predict the occurrence of obesity and cardiovascular diseases at later ages (35). Weight gain and changes in body composition are also observed in postmenopausal women. Assuming that initially high FSH could also affect the BMI and metabolic parameters at later ages, we compared the values at the first visit with those after 10 years. Initially high FSH values were not associated with poorer metabolic parameters, possibly because of the high percentage of women with HRT.

Loss of ovarian function at menopause is associated with changes in lipoprotein patterns, such as increasing concentrations of TG, LDL-C and Lipoprotein a (36, 37). HRT has been shown to have beneficial effects on the atherogenic lipid profile in postmenopausal women by significantly decreasing the concentrations of Lipoprotein a, cholesterol and LDL-C (25). Further studies have also demonstrated a beneficial effect on BMI and body composition (24). Similarly to findings in postmenopausal women, sex hormone administration has been shown to increase fat free mass and the physical fitness of women with TS (38).

Serum TG and LDL-C concentrations are higher in women with TS than in women with 46,XX (39). In our study, we found no significant increase in lipids, possibly because of the favorable effects of hGH treatment. No significant alterations in cholesterol, HDL-C concentrations and TG attributable to HRT were found, in line with previous observations (no changes in lipids during a follow-up of 6 months) (9).

To date, some debate remains regarding the optimal dose, route and type of HRT and the age at initiation. In our study, HRT was administered to 84,4% of all patients. The clinical criterion for starting HRT was detection of hypergonadotropic gonadotropins. Girls started estrogen replacement at a mean age of 164.4 ± 4.54 months. Thus, the age was slightly higher than the current clinical practice guidelines (start between 132–144 months of age), possibly because individual factors (e.g., the desire of the patients to start later) might have influenced the start time. In the follow-up, most girls received oral HRT, and only two patients (for cardiovascular reasons) chose dermal application. Re-analyzing the effects omitting these patients on transdermal HRT resulted only in minor changes of descriptive statistics. Significance did not change (data not shown). Unfortunately, in 7.4% of the cases, HRT was stopped in the follow-up (all after 18 years of age), mainly because of lack of compliance and fear of cancer. However, studies have shown that the risk of breast cancer is low in TS, and long-term treatment with HRT does not appear to induce breast cancer (40, 41). Thus, more efforts must be made to improve adherence.

### Strengths and Limitations of the Study

The main strength of our study is that we had a relatively homogeneous cohort in terms of demographic characteristics (age, BMI, height, and IGF-1) and a long follow-up. However, the study has some limitations. First, the sample size was relatively small. Additionally, owing to the retrospective study design, some data were incomplete, particularly for hormone concentrations (AMH). Gonadotropins were documented in all cases. Another limitation is the change of the assays during the follow-up (20 years), with higher sensitivity assays used in later periods for all metabolic parameters investigated. Additionally, the recommendations of the current clinical practice guidelines could not be considered in most cases, because they were not published until 2016, whereas our study started in 2000. This timing explains why the median age at the start of hGH treatment and HRT differed from the current recommendations, which have now been adopted. Nevertheless, the treatment was offered to large portion of girls with TS treated at our department, and only a few patients stopped treatment prematurely. The different mean age at onset of hGH treatment and HRT and the overlap of the therapies are further limitations. Even in the analysis of the effects with a generalized mixed model, the age-related or endogenous effects (e.g., concerning BMI) could not be excluded completely.

This study illustrates the effects of hGH treatment and HRT in modulating the metabolic profile, even in young girls. Unfavorable effects of hGH on the development of hyperinsulinemia should be monitored and may be used to guide decisions concerning the duration of hGH treatment.

## Data Availability Statement

The raw data supporting the conclusions of this article will be made available by the authors, without undue reservation.

## Ethics Statement

The studies involving human participants were reviewed and approved by Research Ethics Committee of the Hamburg Medical Board. Written informed consent to participate in this study was provided by the participants’ legal guardian/next of kin.

## Author Contributions

SES drafted the manuscript, participated in data analysis and interpreted the results. SGS analyzed and interpreted the results. C-JP revised the manuscript and provided editorial support. WB participated in data collection. FN revised the manuscript and provided editorial support. All authors contributed to the article and approved the submitted version.

## Conflict of Interest

C-JP was employed by Endokrinologikum, Hamburg, Germany. SES and FN were employed by amedes experts GmbH, Hamburg, Germany. WB is employed by MVZ MediVision Altona GmbH.

The remaining author declares that the research was conducted in the absence of any commercial or financial relationships that could be construed as a potential conflict of interest.
